# Effects of Interferometric Radar Altimeter Errors on Marine Gravity Field Inversion

**DOI:** 10.3390/s20092465

**Published:** 2020-04-27

**Authors:** Xiaoyun Wan, Shuanggen Jin, Bo Liu, Song Tian, Weiya Kong, Richard Fiifi Annan

**Affiliations:** 1 School of Land Science and Technology, China University of Geosciences (Beijing), Beijing 100083, China; wanxy@cugb.edu.cn (X.W.); tiansong007@foxmail.com (S.T.); richannan@outlook.com (R.F.A.); 2 Shanxi Key Laboratory of Resources, Environment and Disaster Monitoring, Jinzhong 221166, China; 3 School of Remote Sensing and Geomatics Engineering, Nanjing University of Information Science and Technology, Nanjing 210044, China; 4 Shanghai Astronomical Observatory, Chinese Academy of Sciences, Shanghai 200030, China; 5 Qian Xuesen Laboratory of Space Technology, Beijing 100094, China; liubo@qxslab.cn (B.L.); kongweiya@qxslab.cn (W.K.)

**Keywords:** wide-swath altimetry, vertical deflections, gravity anomaly, relative height, phase error

## Abstract

The traditional altimetry satellite, which is based on pulse-limited radar altimeter, only measures ocean surface heights along tracks; hence, leads to poorer accuracy in the east component of the vertical deflections compared to the north component, which in turn limits the final accuracy of the marine gravity field inversion. Wide-swath altimetry using radar interferometry can measure ocean surface heights in two dimensions and, thus, can be used to compute vertical deflections in an arbitrary direction with the same accuracy. This paper aims to investigate the impact of Interferometric Radar Altimeter (InRA) errors on gravity field inversion. The error propagation between gravity anomalies and InRA measurements is analyzed, and formulas of their relationship are given. By giving a group of possible InRA parameters, numerical simulations are conducted to analyze the accuracy of gravity anomaly inversion. The results show that the accuracy of the gravity anomalies is mainly influenced by the phase errors of InRA; and the errors of gravity anomalies have a linear approximation relationship with the phase errors. The results also show that the east component of the vertical deflections has almost the same accuracy as the north component.

## 1. Introduction

Since the first altimeter named Skylab was launched in 1973, altimetry technology has been applied in several satellite missions by National Aeronautics and Space Administration (NASA) and the European Space Agency (ESA), such as Geosat, Topex/Poseidon, Jason 1 and 2, etc. This is because altimetry plays a significant role in earth sciences. One of the main products provided by altimetry satellite missions is marine gravity field data, which is used widely in geodesy, geophysics, and marine science research [1,2].

Methods of gravity inversion using satellite altimetry observations mainly include the direct method and vertical deflection method. The direct method inverses gravity signal using geoid information, which is directly derived from ocean surface height observations [3,4]. Its shortcoming is the difficulty in removing various environmental noises and mean dynamic topography (MDT) to obtain highly accurate geoid information. Some payloads, such as radiometer and geophysical models, can provide information on these corrections [5]. The vertical deflection method firstly derives vertical deflections by computing ocean surface height differences along the satellite orbits, and then uses these vertical deflections to compute gravity anomalies through the inverse Vening Meinesz formula [6,7]. The advantage of this method is that, the computation of along-track difference can reduce system noises and, consequently, improve the accuracy of gravity field products [8]. Vertical deflections can also be used to derive gravity disturbance through Fast Fourier Transform (FFT) [1,9,10] and recover gravity anomaly by remove-restore method. Using the above approaches, Sandwell and Smith [1,9], Hwang et al. [7], and Andersen and Knudsen [11] published highly accurate global and regional gravity anomaly products, some of which have accuracy as high as 2 mGal [12]. These products have been applied in many fields, such as tectonic structure research [13], bathymetry inversion [14,15,16], and ultra-high degree gravity field modeling [17].

Although the traditional altimetry satellite—which uses the pulse-limited radar altimeter as its main payload—plays a significant role in marine gravity field modeling, it still has some shortcomings. For instance, in the vertical deflection method, the difference computation is usually conducted along-track because the traditional satellite altimetry measures ocean surface heights along-track; thus, difference computation along-track can largely reduce system errors. However, this is not the case for cross-track difference computation. In order to observe the whole earth surface, the inclinations of altimetry satellites are usually designed to be close to 90°. This results in the along-track direction to be close to the north direction, which leads to about three times higher accuracy of north component of vertical deflections compared to the east component [1,18]. Another issue is that the traditional altimeter only measures the elevation of the sub-satellite point, and the data acquisition points are sparse for high-precision differential calculation in different directions. This leads to the difficulty of deriving gravity gradient data using traditional altimetry observations. 

CyroSat [19] mission developed a new altimeter called Synthetic Aperture Radar (SAR) altimeter, which improves along-track resolution compared to traditional altimeter by a factor of 20 [19,20]. Due to its high spatial resolution, accuracy of the derived gravity is twice that of the traditional altimetry satellite observations [12,13]. Correspondingly, it improves the accuracy of vertical gravity gradients derived from its altimetry observations [13,21]. These achievements prove the importance of the initial spatial sampling of altimetry observations. However, it is still difficult to conduct the difference computation with high spatial resolution in cross-track direction. This is because this technology still only measures sea surface height at the sub-satellite points. Due to the inclination being close to 90°, it is still not easy to obtain highly accurate east component of vertical deflections and full tensors of gravity gradients, i.e., $V_{xx}$, $V_{yy}$, $V_{zz}$, $V_{xy}$, $V_{xz}$, $V_{yz}$. Since the vertical deflections are further used to derive gravity anomaly, the poor accuracy of the east component of vertical deflections would limit the accuracy of gravity anomaly.

Currently, a new altimetry called wide-swath altimetry [22], i.e., small angles of interferometric radar [23,24,25], has been developed to overcome the weaknesses of traditional altimetry. This technology can measure ocean surface heights both in along-track and cross-track directions simultaneously [23,24]. Due to the large number of data points, the sea level elevation data obtained by this technology is very helpful for differential calculation. According to Fu et al. [23], the accuracy of sea level elevation measurements provided by this technology can theoretically reach several centimeters at a resolution of several kilometers. Tiangong II, the Chinese space laboratory, has experimented this technology. A simulation based on the parameters of this mission shows that the accuracy of the height measurements is 7 cm on a 5-km grid [24]. However, the accuracy of the actual data is worse due to some system errors, which are difficult to remove. Although the data provided by this mission are not accurate enough for gravity field inversion, not only because of the system errors but also because of the limitation of system parameters, such as the insufficient length of the baseline, it has shown great potential in spatial resolution that has never been realized before [24]. 

As of now, although wide-swath altimetry for ocean height observation is not a matured technology, it has the large potential of resolving the problem of lower accuracy in east component of vertical deflections, as well as the computation of full tensors of gravity gradients with high accuracy. The Surface Water and Ocean Topography (SWOT) mission [23,26], developed by NASA and Centre National d’Etudes Spatiales (CNES), will be launched in 2021. The main scientific objectives of this mission are to understand the oceanic mesoscale, sub-mesoscale processes, and terrestrial hydrology [23,27]. Many simulations have been conducted to evaluate the performance of this mission, especially on the topic of its main scientific objectives [28,29]. Esteban-Fernandez [30] analyzed the overall performance of this mission by considering the kinds of error sources, including systematic errors, random errors, etc. However, it seems its performance in gravity inversion has not yet been considered much. The reason could be that marine gravity field detection is not the main scientific objective of the SWOT mission [23]. Hence, it is imperative to analyze the potential of interferometric radar altimeter (InRA) on gravity field inversion, especially the possible accuracy of gravity products derived by this new kind of altimetry. In addition, the utmost concern in marine gravity field inversion is the accuracy of relative heights (mostly in a small area) if the vertical deflection method is used, but not absolute height accuracy, which is usually given. Due to the close distances in a small area, many system errors are easily reduced in theory, which may reduce system requirements. Thus, the objective of this study is to analyze accuracy of gravity anomaly inversion using InRA observations. 

## 2. Theory and Methods

### 2.1. Radar Interferometry

**Figure 1:**
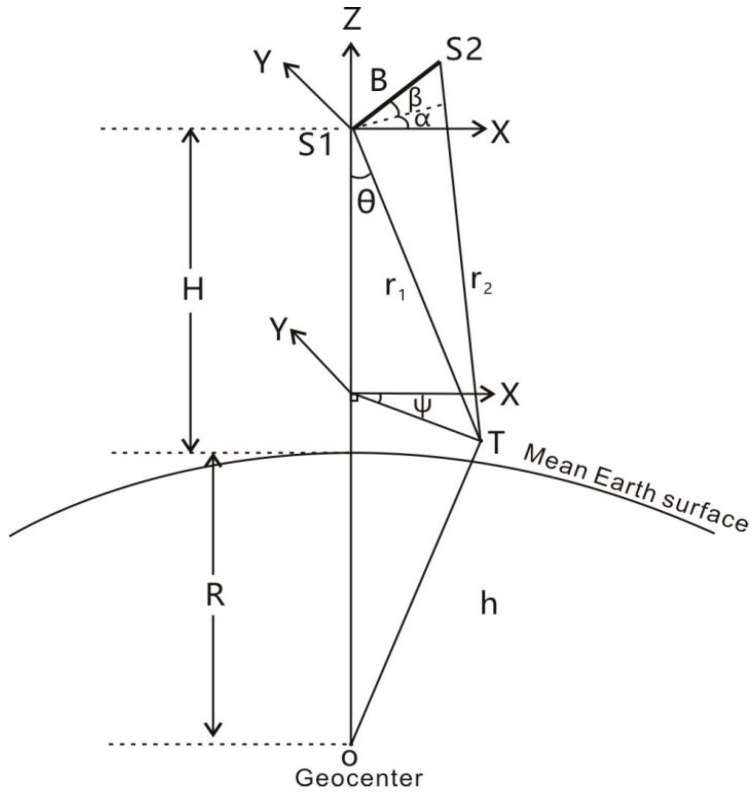
Principle of Interferometric Radar Altimeter (InRA).

The principle of InRA is shown in Figure 1.

In Figure 1, s1 and s2 represent the locations of radar antennas, H represents the height of point s1 above the mean earth surface, R is the radius of the earth, and h represents distance between point T and the geocentric point, denoted as o. At point s1, a Cartesian coordinate system is established, where the X-axis points to the range direction and Y-axis points to the azimuth direction. The Z-axis points to the direction from geocentric point o to point s1. B is the baseline; α, β are respectively, azimuth angle and pitch angle of the baseline. Moreover, θ is the incidence angle, ψ is the azimuth angle of the target T and is also the dihedral angle between o-S1-X and o-S1-T. $r_{1}$ is the slant distance between antenna position s1 and target position T. $r_{2}$ is the slant distance between position of antenna s2 and target T. Hence, the coordinates of s1, s2, and T are (0,0,0),(Bcosβcosα,Bcosβsinα,Bsinβ) and $\left(r_{1}\sin\theta \cos\psi ,r_{1}\sin\theta \sin\psi ,-r_{1}\cos\theta \right)$, respectively. Thus, the relationship between $r_{1}$ and $r_{2}$ can be derived as:

(1) $$r_{2}=\sqrt{B^{2}+r_{1}{}^{2}+2Br_{1}(\sin\beta \cos\theta -\cos\beta \sin\theta \cos(\alpha -\psi ))}$$

The difference between the ranges from the two antennas to the target T is

(2) $$\Delta r=r_{1}-r_{2}=\frac{\lambda }{2\pi }\Delta \Phi$$

where ΔΦ is interferometric phase given by InRA observations and λ is the radar wavelength. The geocentric distance of the target is:

(3) $$h=\sqrt{{\left(H+R\right)}^{2}+r_{1}{}^{2}-2Hr_{1}\cos\theta }$$

where the sum of H and R equals the distance between points s1 and o. Based on Equations (1) and (2), Equation (4) is given as:

(4) $$r_{1}-\frac{\lambda }{2\pi }\Delta \Phi =\sqrt{B^{2}+r_{1}{}^{2}+2Br_{1}(\sin\beta \cos\theta -\cos\beta \sin\theta \cos(\alpha -\psi ))}$$

Based on Equations (3) and (4), h can be obtained using $H,B,\lambda ,\alpha ,\beta ,\psi ,\Delta \Phi ,r_{1}$ and then the absolute height of target T can be represented as h−R. Note that H can be provided by satellite orbit determination. B,λ, α,β,ψ are parameters of interferometric radar and satellite attitude determination;ΔΦ and $r_{1}$ are from interferometric radar observations. According to references [31,32,33], the relative heights between adjacent points can be written as:

(5) $$\Delta h=\frac{\lambda r\sin\theta }{B_{⊥}}\frac{\Delta \varphi }{2\pi }$$

where $\Delta h=h_{1}-h_{2}$, $h_{1}$, and $h_{2}$ denote heights of the adjacent points and r is the slant distance. θ denotes the incidence angle;Δφ is the phase difference between the two points, i.e., $\Delta \varphi =\Delta \Phi_{1}-\Delta \Phi_{2}$ where $\Delta \Phi_{1}$ and $\Delta \Phi_{2}$ denote interferometric phases of the adjacent points, and $B_{⊥}$ is the orthogonal baseline, which can be calculated as [33]:

(6) $$B_{⊥}=B(\sin\beta \sin\theta +\cos\beta \cos\theta \cos(\alpha -\psi ))$$

### 2.2. Influence of Phase Error on Gravity Field Inversion

According to the above section, the sea surface heights (*SSH*) can be obtained from InRA observations, i.e., the differences between h and the distance from the equipotential surface of the reference ellipsoid to the earth center [34,35]. Before being used in gravity field computations, *SSH* should be processed for several corrections [5,36] as given in Equation (7):

(7) SSH=N+MDT+C

where N represents geoid heights, MDT is the mean dynamic topography and C represents geophysical corrections [5] (i.e., ionospheric correction, dry and wet tropospheric correction, sea sate correction, tide corrections, etc.). Based on geoid difference computation derived from Equation (7), vertical deflections can be obtained as [1,37]:

(8) $$\left\{ \begin{array}{l} \varepsilon =\frac{dN}{dx}=\frac{dN}{Rd\vartheta }\\ \eta =\frac{dN}{dy}=\frac{dN}{R\sin\vartheta d\phi } \end{array}\right.$$

where ε is north component of vertical deflections and η is east component of vertical deflections. ϑ and ϕ are the co-latitude and longitude of the computing points, respectively. Obviously, the vertical deflections are highly correlated with relative heights of the ocean surface; hence, as long as the vertical deflections are obtained, gravity anomalies can be derived as [6,7]:

(9) $$\Delta g=-\frac{\gamma }{4\pi }\iint_{\delta }H\prime \left(\varepsilon \cos A+\eta \sin A\right)d\delta$$

where [6]

(10) $$H\prime =\left(-\frac{\cos\frac{\omega }{2}}{2\sin^{2}\frac{\omega }{2}}+\frac{\cos\frac{\omega }{2}(3+2\sin\frac{\omega }{2})}{2\sin\frac{\omega }{2}(1+\sin\frac{\omega }{2})}\right)$$

and A is the azimuth from the integral point to the query point. ω is the sphere distance between the two points. The integration can be conducted in the space or spectral domain [1,6]. However, in order to minimize the influence of computational errors, the integration process is not incorporated in this study. This is because such calculations may result in computational errors, such as the innermost zone effect, and it would be difficult to distinguish which errors originate from the integration process or from the InRA observations. Instead, the relationship between the accuracy of vertical deflections and gravity anomalies [38,39] is used directly to discuss the influence of InRA errors on gravity field inversion as

(11) $$\delta_{\Delta g}^{2}=\gamma^{2}\left(\delta_{\varepsilon }^{2}+\delta_{\eta }^{2}\right)$$

where γ is the normal gravity, $\delta_{\Delta g}^{}$, $\delta_{\varepsilon }^{}$ and $\delta_{\eta }^{}$ denote errors of Δg, ε,η, respectively. According to Equations (5) and (11), the relationship between precision of gravity anomaly and interferometric phase can be derived as:

(12) $$\delta_{\Delta g}^{}=\frac{\gamma \lambda H\tan\theta }{\pi B_{⊥}l}\delta_{\Delta \Phi }$$

where l is the spherical distance used for vertical deflection computation and $\delta_{\Delta \Phi }$ denotes phase error.

## 3. Numerical Analysis and Results

**Figure 2:**
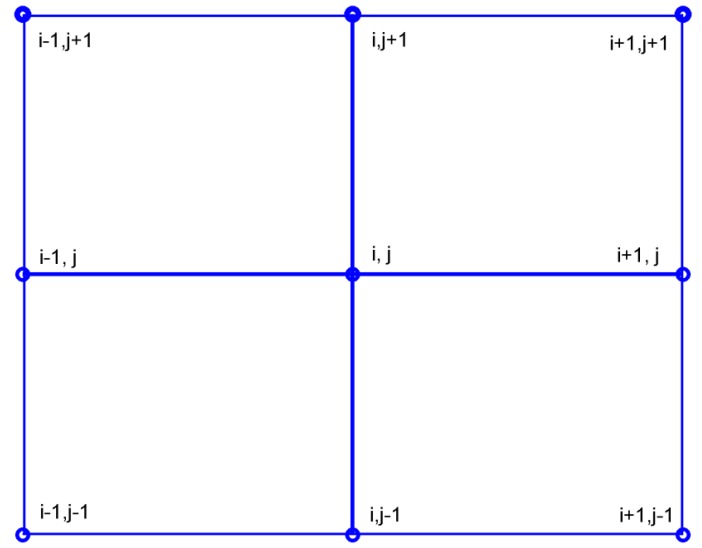
Point distribution.

The height accuracy is the basis for discussion in gravity field product accuracy. For convenience, we define two kinds of height accuracy, i.e., absolute accuracy and relative accuracy. In this study, the relative height is the average elevation difference between a simulated elevation point and its four adjacent points. The relative height accuracy is evaluated by the relative height differences between the results with and without radar interferometer parameter errors, denoted as $\sigma_{rel}$. The absolute elevation errors refer to the elevation differences between simulated sea level elevation points without InRA errors and corresponding elevation points with InRA errors, denoted as $\sigma_{abs}$. In order to derive the formulas for computing $\sigma_{rel}$(see Equation (13)) and $\sigma_{abs}$(see Equation (14)), the points distribution is given in Figure 2.

(13) $$\begin{array}{l} \sigma_{rel}=\left\{h^{\prime }(i,j)-\frac{h^{\prime }(i+1,j)+h^{\prime }(i-1,j)+h^{\prime }(i,j+1)+h^{\prime }(i,j-1)}{4}\right\}\\ \;-\left\{h(i,j)-\frac{h(i+1,j)+h(i-1,j)+h(i,j+1)+h(i,j-1)}{4}\right\} \end{array}$$

(14) $$\sigma_{abs}=h^{\prime }(i,j)-h(i,j)$$

where h is the assumed true sea surface height (i.e., without adding interferometric errors) and $h^{\prime }$ denotes the sea surface height affected by interferometric errors. (i,j) is the coordinate of the query point and h(i,j) refers to the height of point (i,j). The vertical deflections at point (i,j) are calculated as follows,

(15) $$\left\{ \begin{array}{l} \varepsilon_{i,j}=\frac{h(i,j+1)-h(i,j-1)}{l_{1}}\\ \eta_{i,j}=\frac{h(i+1,j)-h(i-1,j)}{l_{2}} \end{array}\right.\;$$

or

(16) $$\left\{ \begin{array}{l} \varepsilon_{i,j}^{\prime }=\frac{h^{\prime }(i,j+1)-h^{\prime }(i,j-1)}{l_{1}}\\ \eta_{i,j}^{\prime }=\frac{h^{\prime }(i+1,j)-h^{\prime }(i-1,j)}{l_{2}} \end{array}\right.$$

where

(17) $$\left\{ \begin{array}{l} l_{1}=R\left(\vartheta_{i,j+1}-\vartheta_{i,j-1}\right)\\ l_{2}=R\cos\left(\vartheta_{i,j}\right)\left(\phi_{i+1,j}-\phi_{i-1,j}\right) \end{array}\right.$$

R is the radius of the earth;$\vartheta_{i,j+1}$, $\vartheta_{i,j}$, $\vartheta_{i,j-1}$ are the co-latitudes of points (i,j+1), (i,j) and (i,j−1), respectively; $\phi_{i,j+1}$ and $\phi_{i,j-1}$ are the longitudes of points (i+1,j) and (i−1,j), respectively. The parameters used for simulating sea surface heights are as given in Table 1. 

**Figure 3:**
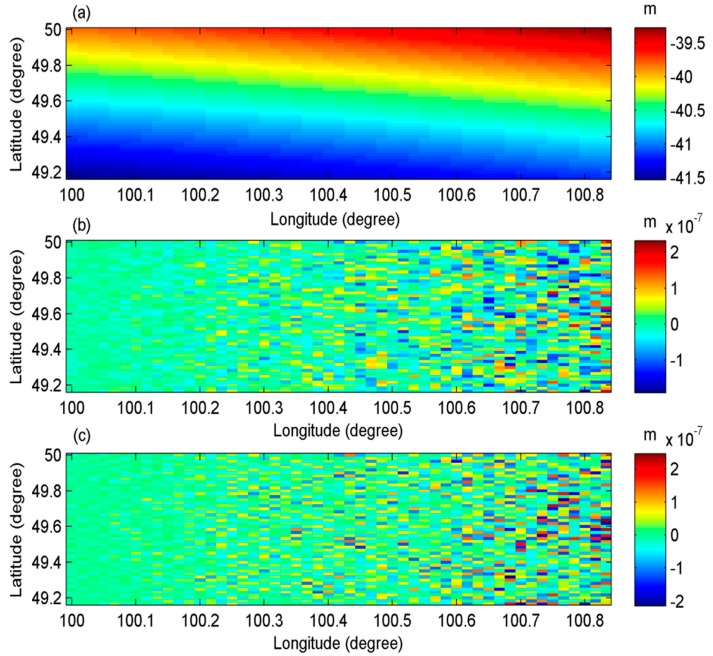
The simulated N and their absolute and relative errors when no interferometric errors are added: (**a**) the simulated N; (**b**) absolute height errors; (**c**) relative height errors.

The satellite is designed to fly from south to north. Hence, the range direction is from the west to the east and the azimuth direction is from the south to the north. The former EGM2008 up to degree/order (d/o) 2–200 [17] was used to simulate geoid (denoted as $N_{0}$), gravity anomaly and vertical deflection data at a resolution of 1′ × 1′ in an area with latitude ranging from 49.17°~50° and longitude ranging from 100°~100.83°. The geoid, gravity anomaly and vertical deflections data simulated by EGM2008 are used as the true values. Since the aim of this paper is to discuss the influence of InRA errors on gravity field recovery, geoid values are used as sea surface heights directly although mean dynamic topography also influence the accuracy of gravity field inversion. Based on Equations (3)–(4), $r_{1}$ and ΔΦ are firstly derived using $N_{0}$, B, H, α, β and ψ. These values are seen as InRA observations. In the second step, new geoid data denoted as N, can be derived using InRA observations and InRA parameters. In this step, InRA errors such as errors of B, H, α, β, ψ, and ΔΦ are added, which results in errors in the newly derived geoid data and the finally inverted gravity field. It needs to be noted that environmental influences [5,36], such as atmospheric effects, also influence the accuracy of gravity field detection. However, if all these factors are considered, it would be difficult to distinguish which errors originate from the InRA system and which errors originate from environment corrections. Hence, in this study, the environmental influences are not considered and will be investigated in future studies. Obviously, if no errors are added, N should be equal to $N_{0}$. However, due to the computational errors, there are minor differences between N and $N_{0}$ (see Figure 3). In Figure 3, most of the errors are smaller than 10^−7^ m. Thus, N was used to compute vertical deflections, which were compared with the assumed true values computed directly from EGM2008. The related statistics of their differences are shown in Table 2, which outlines the reliability of the method for computing vertical deflections in this study. 

According to Table 2, if no interferometric errors are added, the mean and standard deviation (std) of the two components of vertical deflections are both smaller than 0.005s. Correspondingly, according to Equation (13), the mean and standard deviation of the gravity anomaly errors are about 0.026 mGal and 0.015 mGal, respectively. 

**Figure 4:**
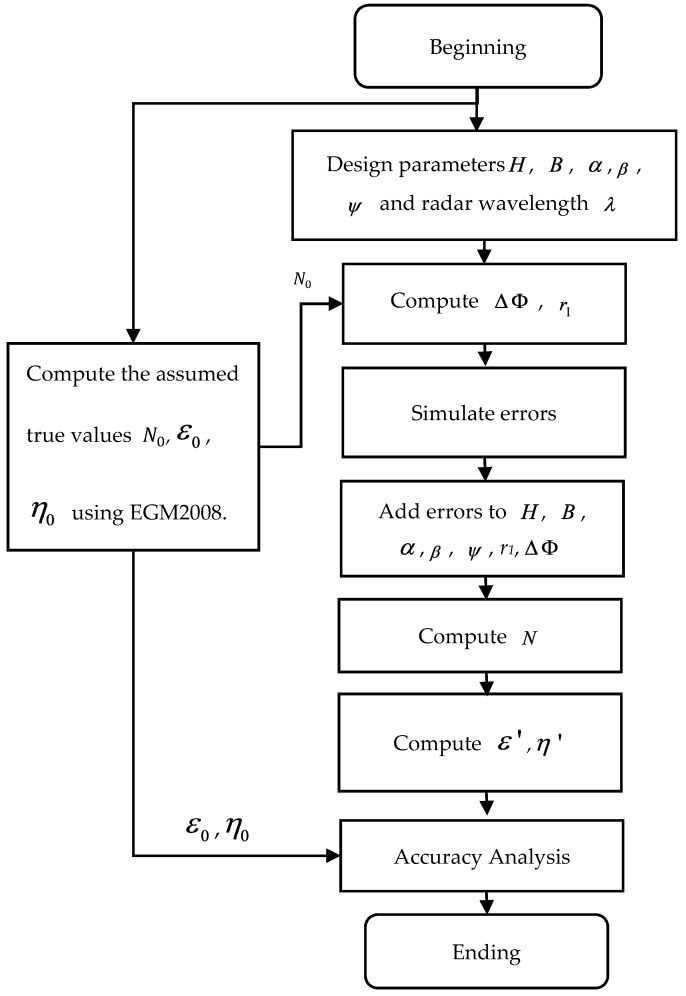
Computational process.

In order to analyze the influences of B, H, α, β, ψ, $r_{1}$, and ΔΦ on the inversion of vertical deflections and gravity anomalies, noise (Table 3) is added to the interferometric system, which is similar in design to the SWOT mission, and geoid data are obtained. Vertical deflections are then computed using Equation (16). By comparison with the assumed true values (from EGM2008 directly), one can evaluate the effects of different magnitudes of the InRA parameter errors on vertical deflections and gravity anomaly inversion. It needs to be noted that what is of interest in computing gravity filed products is relative heights. According to Jin et al. [40], and González and Bräutigam [32], apart from interferometric phase errors, other InRA errors can be seen as systematic errors in a small area. Hence, apart from interferometric phase errors, the errors shown in Table 3 are the same for computing vertical deflections in a small area. The interferometric phase errors are added as white noise with standard deviation as given in Table 3. A schematic view of the computational process is shown in Figure 4. 

By adding the errors given in Table 3, the influences of different InRA parameter errors on absolute height and relative height are evaluated by Equations (13) and (14). The results are as given in Table 4 and Table 5. 

In Table 4 and Table 5, the second columns give the error std values of the parameters listed in the first column. The third, fourth, and fifth columns represent mean, std, and max values of the absolute height or relative height errors caused by the parameter errors. According to these tables, the height errors caused by InRA baseline errors, satellite height errors, attitude errors and slant distance errors can be reduced largely when computing relative heights. For example, the influences of orbit height errors (i.e., 2 cm in this simulation) can also yield same level of absolute height errors for the ocean surface measurements but very small errors for the relative heights. This is because, for the adjacent points, the influence of orbit height errors is almost the same and difference computation can reduce them. Since the relative heights are the input data for computing vertical deflections, baseline errors, satellite height errors, attitude errors and slant distance errors would not be the dominant error sources for vertical deflections.

The accuracy of vertical defections is evaluated by the comparison between values from Equation (14) and the assumed true values from EGM2008, i.e., $\varepsilon_{0}$, $\eta_{0}$ in Figure 4. Finally, the gravity anomaly accuracy is evaluated by Equation (11). The statistics of results for vertical deflections and gravity anomaly are shown in Table 6, Table 7 and Table 8.

It is conspicuous from Table 6, Table 7 and Table 8 that the phase errors limit the accuracy of vertical deflections and gravity anomaly. The reason is that the height errors caused by interferometric phase errors are different even for the adjacent points, and the use of numerical method, such as difference computation to reduce these errors, is difficult. This is evident in the results, where the magnitudes of absolute height errors and relative heights errors are almost the same. 

From the above analysis, for gravity, and even gravity gradient inversion using InRA observations, it is superior to firstly compute vertical deflections and then use them to derive gravity anomalies [6] or gravity disturbance [1], considering that as the influences of InRA baseline errors, satellite height errors, attitude errors and slant distance errors can be reduced during the vertical deflections computation. The interpolation and difference computation algorithms should be studied further to improve accuracy of vertical deflection computation. Methods of [41,42] may be helpful on this topic.

It also needs to be pointed out that in this simulation, the value of B is set to 50 m and not 10 m as in SWOT. This means if the value of B is changed to 10 m and other parameters in Table 1 and Table 3 are not changed, the std of the derived gravity anomaly would be magnified 5 times, according to Equations (12) and (6), to 19.55 mGal. Definitely, such accuracy is poor. However, these are derived for the gravity anomaly with size of 1′ × 1′. For larger size, such as 5′ × 5′, the accuracy would be 3.91 mGal.

## 4. Discussion

**Figure 5:**
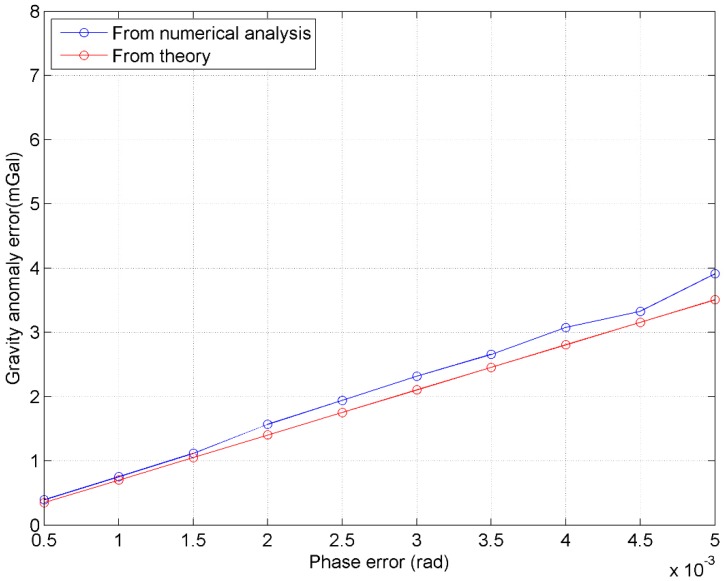
Standard deviations of gravity anomaly errors in the case of different phase errors.

According to the above results and analysis, the phase accuracy is the key to improving the accuracy of the gravity field recovery using InRA observations. In order to show the relationship between phase errors and gravity anomaly accuracy, numerical tests are further conducted with the phase errors reset from 0.0005 to 0.005 rad at an equal interval of 0.0005 rad while maintaining the other parameters given in Table 1 and Table 3. The standard deviations of the final gravity anomaly errors are shown in Figure 5.

In Figure 5, ‘From numerical analysis’ denotes gravity anomaly errors derived from the simulations (see Figure 4) and ‘From theory’ denotes those values derived by Equation (18), which are derived from Equations (11) and (16), based on the InRA parameters used in this paper.

(18) $$\delta_{\Delta g}^{}=\frac{\gamma \lambda H\sin\theta \sqrt{l_{1}^{2}+l_{2}^{2}}}{2\pi Bl_{1}l_{2}\cos^{2}\theta }\delta_{\Delta \Phi }$$

Please note $l_{1}\approx 3.7106$ km and $l_{2}\approx 2.4058$ km in this study. According to Table 1, θ changes from 0.5°~4.8325°. In order to plot Figure 5, mean value of θ is used to compute $\delta_{\Delta g}^{}$ by using Equation (18). According to Figure 5, gravity anomaly errors have a nearly linear relationship with InRA phase errors, which validates Equations (12) and (18). In order to obtain gravity anomalies with high accuracy, wide-swath altimetry systems should have high interferometric phase accuracy, which can be achieved by multi-looking [24,41]. Multi-looking reduces phase error by averaging a squared number of pixels. The more pixels averaged by multi-looking, the smaller the altimetry error; however, spatial resolution will be lowered in the process. If the vertical deflection method is used to compute gravity anomalies, the final accuracy is mainly controlled by relative height accuracy, and the relative height accuracy of InRA is mainly controlled by phase errors. Consequently, if the accuracy target of gravity anomalies is to be better than 1 mGal under the parameters of InRA system set in this paper, then the phase error should be smaller than 1.5 mrad.

**Figure 6:**
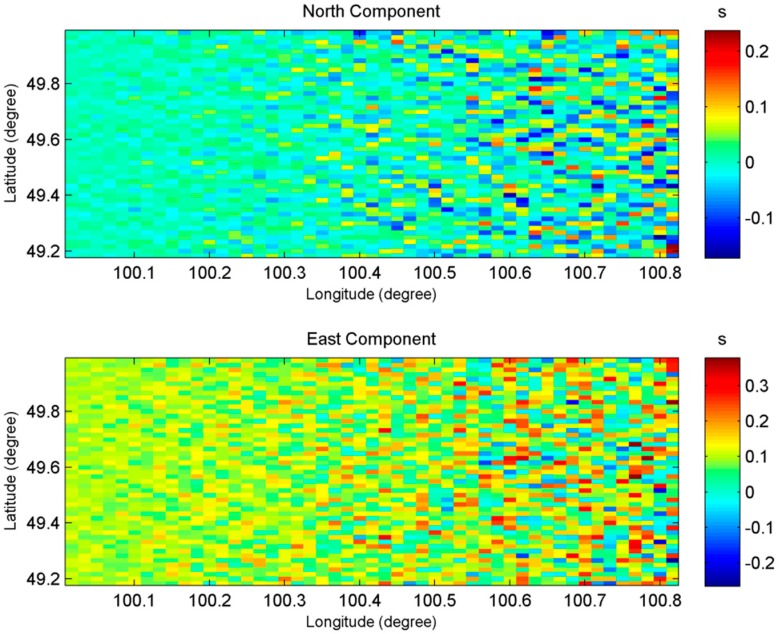
Vertical deflection errors when phase error is 0.5 mrad.

**Figure 7:**
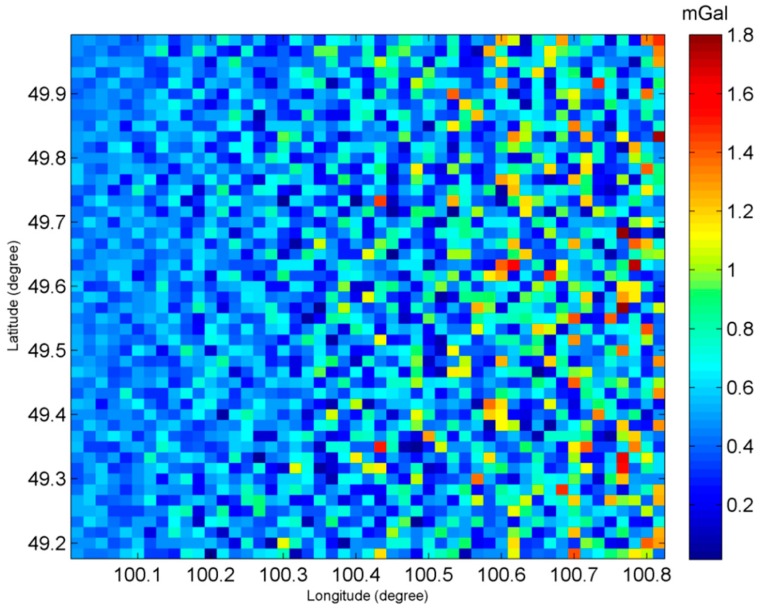
Gravity anomaly errors when phase error is 0.5 mrad.

In order to analyze the error distribution in space domain, as an example, Table 9 gives the accuracy statistics of gravity anomalies when phase error is 0.5 mrad and the error distribution in space is shown in Figure 6 and Figure 7.

It can be observed from Table 9 that, the two components of vertical deflections both have high accuracy, which shows the superiority of InRA as compared to the traditional altimetry. It can compensate for the weakness (lower accuracy in the east direction than in the north direction) of the vertical deflections calculated by traditional altimetry satellites based on pulse-limited radar altimeter. Since the computation of gravity anomalies require east and north components of vertical deflections, an equally accurate measurement of vertical deflections in both directions will contribute to the overall accuracy improvement of gravity anomalies. It needs to be pointed out from Table 9 that, there are system errors. For example, mean gravity anomaly error is 0.45 mGal, but not close to 0. This is mainly caused by the error of *β*, which is evident in Table 8.

**Figure 8:**
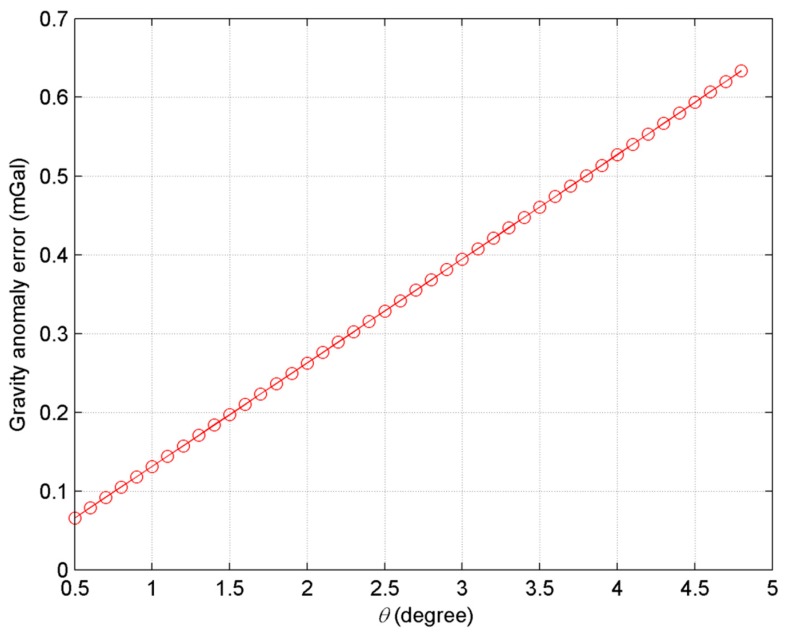
Gravity anomaly errors with different θ when phase error is 0.5 mrad.

In addition, according to Figure 6 and Figure 7, the farther the sub-satellite points, the worse the accuracy of vertical deflections and gravity anomalies is. This is consistent with Equation (18) since $\frac{\sin\theta }{\cos^{2}\theta }$ is larger in far range than it is in the near range. In order to prove this point, the gravity anomaly errors with different θ are shown in Figure 8 when $\delta_{\Delta \Phi }$ = 0.5 mrad.

According to Figure 8, gravity anomaly errors in the far range (e.g., $\theta >4.5^{{}^{\circ}}$) are about 6~10 times higher than those in the near range (e.g., $\theta =0.5^{{}^{\circ}}$). For example, the gravity anomaly error is 0.634 and 0.066 mGal at $\theta =4.8^{{}^{\circ}}$ and $\theta =0.5^{{}^{\circ}}$, respectively. In Kong et al. [24], it was pointed out that the phase accuracy in the far range should be 10 times better than that in the near range in order to obtain same accuracy of ocean surface height. Therefore, the incidence angle should not be large if highly accurate gravity field products are to be obtained.

**Figure 9:**
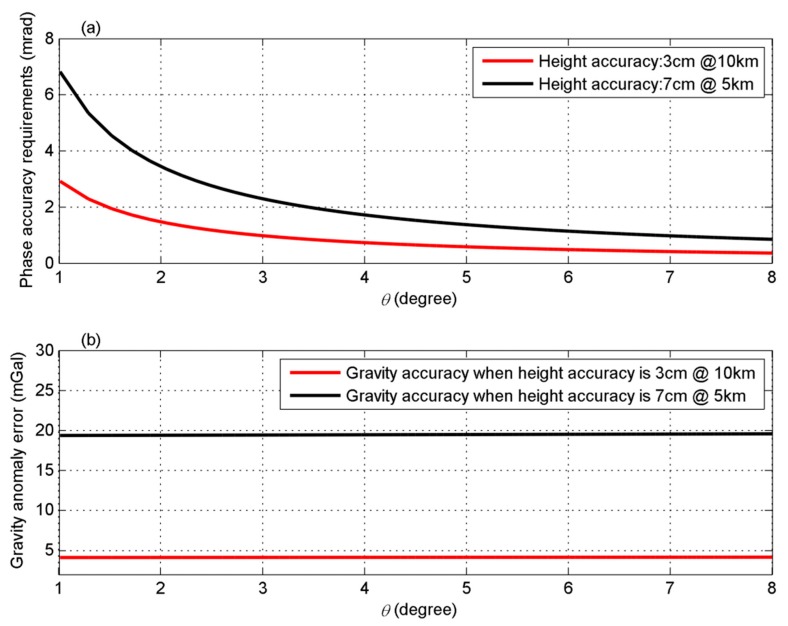
Phase accuracy requirements (**a**) and gravity anomaly errors (**b**) with different height accuracy and *θ*.

Finally, as already mentioned, phase errors can be reduced by multi-looking [43]. The phase errors would be different if the sizes of the grid are different, because different numbers of the observations can be used to compute mean values of the area. In other words, the accuracy of the ocean surface heights would be different if the spatial resolution is different. For example, according to parameters of InRA loaded in Tiangong II, the height accuracy should be better than 7 cm on a 5-km grid but 3 cm on a 10-km grid [24]. Considering the parameters of Tiangong II (see Table 10), Figure 9a represents the phase accuracy requirements for ocean surface height measurements with accuracy of 7 cm on a 5-km grid and 3 cm on a 10-km grid with varying values of θ. The corresponding gravity anomaly errors derived using Equation (18) are also given in Figure 9b. Please note that values of $l_{1}$ and $l_{2}$ equal the size of the grids, i.e., 5 km or 10 km. From Figure 9b, it seems that gravity derived from InRA of Tiangong II will not have higher accuracy and spatial resolution compared to the traditional altimetry products. The main reason could be that some system parameters are limited in the Tiangong II mission, such as the baseline. If length of the baseline is 10 m, the accuracy will be improved by more than 4 times. If Ka-band wave is used, the accuracy would further be improved by nearly 3 times. Furthermore, if longer time of observations and more satellites observations are used, the accuracy could further be improved further. Apart from the effects of InRA parameters, geophysical effects would be the other limitations for marine gravity field recovery, which needs to be studied in the future.

## 5. Conclusions

This research mainly focuses on analyzing the effects of InRA errors on the accuracy of marine gravity field inversion. The following conclusions can be drawn: it is easy to realize equal precision of measurement of vertical deflections by InRA, which will make up for the weaknesses of traditional altimetry satellites (east component of vertical deflection products being inferior to north component). In addition to phase errors, InRA baseline errors, orbit height errors, attitude errors, and slant errors can be greatly reduced by using the vertical deflection method in computing marine gravity field products. Therefore, the final accuracy of gravity anomaly products is mainly affected by phase errors. The accuracy of gravity anomaly products and phase errors satisfy an approximate proportional relationship. In order to obtain highly accurate marine gravity field products by using InRA technology, improving phase accuracy is the key issue. Based on results attained, it can be concluded that it is feasible to use InRA technology to detect highly accurate marine gravity fields. However, the impact of environmental noises on the accuracy of gravity field detection needs to be further investigated with precise InRA observations.

## Figures and Tables

**Table 1:** Altimeter parameters.

Term	Value
H	800 km
B	50 m
α	0°
β	0°
ψ	0°
λ	0.86 cm
θ	0.5°~4.8325°

**Table 2:** Absolute value statistics of computing errors when no interferometric errors are added.

Type	Min	Max	Mean	Std	Relative Error
N **(cm)**	3.54 × 10^−^^10^	2.32 × 10^−^^5^	3.44 × 10^−^^6^	3.17 × 10^−^^6^	0.00001%
ε **(s)**	7.79 × 10^−6^	0.010	0.004	0.002	0.11%
η **(s)**	1.93 × 10^−7^	0.010	0.003	0.002	0.21%

**Table 3:** Parameter errors.

Parameter	Error
B	1 × 10^−5^ m
H	0.02 m
α	0.1 s
β	0.1 s
ψ	0.1 s
$r_{1}$	0.02 m
ΔΦ	0.005 rad

**Table 4:** Statistics of absolute height errors.

Parameters	Error	Mean (m)	Std (m)	Max (m)
*B*	1 × 10^−5^ m	4.21 × 10^−4^	3.35 × 10^−4^	1.14 × 10^−3^
*H*	0.02 m	−2.00 × 10^−2^	4.68 × 10^−8^	−2.00 × 10^−2^
*α*	0.1 s	−5.27 × 10^−10^	4.68 × 10^−8^	2.32 × 10^−7^
*β*	0.1 s	−1.80 × 10^−2^	8.59 × 10^−3^	−3.39 × 10^−3^
*ψ*	0.1 s	−5.27 × 10^−10^	4.68 × 10^−8^	2.32 × 10^−7^
*r_1_*	0.02 m	2.00 × 10^−2^	2.09 × 10^−5^	2.00 × 10^−2^
ΔΦ	0.005 rad	−5.48 × 10^−5^	5.66 × 10^−3^	2.70 × 10^−2^

**Table 5:** Statistics of relative height errors.

Parameters	Error	Mean (m)	Std (m)	Max (m)
*B*	1 × 10^−5^ m	6.02 × 10^−8^	1.93 × 10^−6^	2.08 × 10^−5^
*H*	0.02 m	5.95 × 10^−11^	5.27 × 10^−8^	2.47 × 10^−7^
*α*	0.1 s	5.94 × 10^−11^	5.27 × 10^−8^	2.47 × 10^−7^
*β*	0.1 s	1.62 × 10^−10^	3.94 × 10^−5^	2.94 × 10^−4^
*ψ*	0.1 s	5.94 × 10^−11^	5.27 × 10^−8^	2.47 × 10^−7^
*r_1_*	0.02 m	−3.66 × 10^−9^	1.29 × 10^−7^	2.26 × 10^−7^
ΔΦ	0.005 rad	6.79 × 10^−6^	6.26 × 10^−3^	3.14 × 10^−2^

**Table 6:** Statistics of north component of vertical deflection errors.

Parameters	Error	Mean (rad)	Std (rad)	Max (rad)
*B*	1 × 10^−5^ m	2.14 × 10^−8^	1.13 × 10^−8^	4.92 × 10^−8^
*H*	0.02 m	2.15 × 10^−8^	1.13 × 10^−8^	4.95 × 10^−8^
*α*	0.1 s	2.15 × 10^−8^	1.13 × 10^−8^	4.95 × 10^−8^
*β*	0.1 s	2.42 × 10^−8^	1.14 × 10^−8^	5.38 × 10^−8^
*ψ*	0.1 s	2.15 × 10^−8^	1.13 × 10^−8^	4.95 × 10^−8^
*r_1_*	0.02 m	2.15 × 10^−8^	1.13 × 10^−8^	4.95 × 10^−8^
ΔΦ	0.005 rad	2.25 × 10^−8^	2.14 × 10^−6^	9.54 × 10^−6^

**Table 7:** Statistics of east component of vertical deflection errors.

Parameters	Error	Mean (rad)	Std (rad)	Max (rad)
*B*	1 × 10^−5^ m	−2.69 × 10^−8^	1.96 × 10^−8^	2.65 × 10^−8^
*H*	0.02 m	−8.37 × 10^−9^	1.70 × 10^−8^	3.25 × 10^−8^
*α*	0.1 s	−8.37 × 10^−9^	1.70 × 10^−8^	3.25 × 10^−8^
*β*	0.1 s	4.76 × 10^−7^	1.70 × 10^−8^	5.17 × 10^−7^
*ψ*	0.1 s	−8.37 × 10^−9^	1.70 × 10^−8^	3.25 × 10^−8^
*r_1_*	0.02 m	−7.21 × 10^−9^	1.70 × 10^−8^	3.40 × 10^−8^
ΔΦ	0.005 rad	−1.04 × 10^−8^	3.37 × 10^−6^	1.48 × 10^−5^

**Table 8:** Statistics of gravity anomaly errors.

Parameters	Error	Mean (mGal)	Std (mGal)	Max (mGal)
*B*	1 × 10^−5^ m	0.03	0.02	0.05
*H*	0.02 m	0.02	0.02	0.06
*α*	0.1 s	0.02	0.02	0.06
*β*	0.1 s	0.47	0.02	0.51
*ψ*	0.1 s	0.02	0.02	0.06
*r1*	0.02 m	0.02	0.02	0.06
ΔΦ	0.005 rad	0.02	3.91	17.28
*Total*	/	0.47	3.91	17.29

**Table 9:** Vertical deflections and gravity anomaly errors when interferometric error is 0.5 mrad.

Term	Min	Max	Mean	Std
ε(rad)	−8.34 × 10^−7^	1.15 × 10^−6^	2.53 × 10^−8^	2.17 × 10^−7^
η(rad)	−1.29 × 10^−6^	1.84 × 10^−6^	4.58 × 10^−7^	3.43 × 10^−7^
*△g*(mGal)	−1.51	2.13	0.45	0.40

**Table 10:** Parameter of Tiangong II.

Parameter	Value
B	2.3 m
H	380 km
α	0°
β	5°
ψ	0°
λ	2.21 cm
θ	1.01~8°
